# Long noncoding RNA SNHG12 is a potential diagnostic and prognostic biomarker in various tumors

**DOI:** 10.1186/s41016-021-00250-4

**Published:** 2021-08-09

**Authors:** Xinzhuang Wang, Qiuyi Jiang, Cheng Zhang, Quan Yang, Lixiang Wang, Jian Zhang, Ligang Wang, Xin Chen, Xu Hou, Dayong Han, Jianing Wu, Shiguang Zhao

**Affiliations:** 1 Department of Neurosurgery, First Affiliated Hospital of Harbin Medical University, Harbin, 150001 Heilongjiang Province China; 2 Key Colleges and Universities Laboratory of Neurosurgery in Heilongjiang Province, Harbin, 150001 Heilongjiang Province China; 3 Institute of Neuroscience, Sino-Russian Medical Research Center, Harbin Medical University, Harbin, 150001 Heilongjiang Province China; 4 North Broward Preparatory School, 7600 Lyons Road, Coconut Creek, FL 33073 USA; 5 Department of General Surgery, The First Affiliated Hospital of Harbin Medical University, Harbin, China; 6 Department of Neurosurgery, The Pinghu Hospital of Shenzhen University, Shenzhen, 518100 Guangdong Province China

**Keywords:** Long noncoding RNAs, SNHG12, TCGA, Biomarker

## Abstract

**Background:**

Tumors are the second most common cause of death in humans worldwide, second only to cardiovascular and cerebrovascular diseases. Although methods and techniques for the treatment of tumors continue to improve, the effect is not satisfactory. These may lack effective therapeutic targets. This study aimed to evaluate the value of SNHG12 as a biomarker in the prognosis and clinical characteristics of various cancer patients.

**Methods:**

We analyzed SNHG12 expression and plotted the survival curves of all cancer samples in the TCGA database using the GEPIA tool. Then, we searched for eligible papers up to April 1, 2019, in databases. Next, the data were extracted from studies examining SNHG12 expression, overall survival and clinicopathological features in patients with malignant tumors. We used Review Manager 5.3 and Stata 15 software to analyze the statistical data.

**Results:**

In the TCGA database, abnormally high expression of SNHG12 in tumor samples indicates that the patient has a poor prognosis. Results of meta-analysis is that SNHG12 high expression is related to low overall survival (HR = 2.72, 95% CI = 1.95–3.8, *P* < 0.00001), high tumor stage (OR = 3.94, 95% CI = 2.80–5.53, *P* < 0.00001), high grade (OR = 2.04, 95% CI = 1.18–3.51, *P* = 0.01), distant metastasis (OR = 2.20, 95% CI = 1.40–3.46, *P* = 0.0006), tumor size (OR = 2.79, 95% CI = 1.89–4.14, *P* < 0.00001), and lymph node metastasis (OR = 2.66, 95% CI = 1.65–4.29, *P* < 0.0001).

**Conclusions:**

Our study confirmed that the high expression level of SNHG12 is closely related to the clinicopathological characteristics and prognosis of patients and is a new predictive biomarker for various cancer patients.

**Supplementary Information:**

The online version contains supplementary material available at 10.1186/s41016-021-00250-4.

## Background

According to the Global Burden of Disease Study (GBD), in 2018, there were approximately 18.1 million newly diagnosed tumor patients and 9.6 million patient deaths associated with tumors; malignant tumors are the second most common killer of humans worldwide, second only to cardiovascular and cerebrovascular diseases [1]. Although immunotherapy, nanotechnology, and new technology have been used in the clinic, the curative rates are unsatisfactory. Furthermore, some patients have not shown improvements in quality of life. Due to tumor growth and metastasis [2], unique hallmarks of cancers, and the limited treatment efficacy, there is a clinical need for new diagnostic and prognostic biomarkers to promote early detection and early intervention [3].

Long noncoding RNAs (lncRNAs) are currently a comparatively hot RNA. What is impressive is that it does not develop reading frames and coding capabilities. Compared with approximately 2% of the protein-coding genes, most lncRNAs play a negligible role in transcription and translation [3, 4]. With the emergence of high-throughput sequencing technology and public databases, more and more people study lncRNA. It was gradually recognized that lncRNAs are involved in transcriptional and post-transcriptional regulation by recruiting transcription factors, remodeling chromatin, splicing pre-mRNA, and acting as molecular sponges and scaffolds in multifarious diseases and tumors. These molecules act as tumor promoters or tumor suppressors in the multistep development of human tumors [5, 6, 7]. In addition, lncRNAs can be present in many tissues and fluids for a long time, and can be used as a biomarker for extensive screening of diseases [8].

The US government-sponsored database The Cancer Genome Atlas (TCGA) uses high-throughput genome sequencing, gene chip technology, and multidimensional data integration analysis to collect data on almost all human cancers (the genomic variation and gene expression maps of more than 50 tumors, including subtypes) to provide a resource for the elucidation of the mechanisms of cancer occurrence and development. Based on this database, we used web tools to explore SNHG12 expression in different patient samples in the TCGA database and tried to generate a curve to show the relationship between SNHG12 expression and patient prognosis. In addition, this study provides new biomarkers for clinical diagnosis and treatment to detect tumors early and help select improved treatments.

The long noncoding RNA SNHG12 (small nucleolar RNA host gene 12) was first reported to be upregulated in microarray data in endometrial carcinoma by Zhai. SNHG12 is located at 1p35.3 (chr1: -28905061 - -28909492) and includes 6 exons, which can be translated to generate 8 transcript variants with polyA tails [9]. SNHG12 is always upregulated in the formation and development of the endocrine system, digestive system, reproductive system, locomotor system, and nervous system. In tumors, SNHG12, which is an oncogene, promotes the process of tumor formation through the cell cycle, invasion, metastasis, and apoptosis. Ding et al. showed that lncRNA SNHG12 affects the Wnt/β-catenin signaling pathway to induce cancer cell proliferation and metastasis in thyroid cancer [10]. Liu et al. found that inhibition of the SNHG12-miR-195-SOX5 axis significantly obstructs the malignant biological behavior of glioma cells [11]. Sun et al. noted that lncRNA SNHG12 accelerates the deterioration of patients with ovarian cancer by upregulating SOX4 [12]. Therefore, whether SNHG12 is a biomarker for the detection and treatment of tumors is a growing concern.

SNHG12 was shown to be an effective diagnostic biomarker in nasopharyngeal carcinoma with a multivariate Cox regression analysis [13]. Furthermore, increasing evidence suggests that overexpression of SNHG12 is linked to a poor prognosis or a high risk of clinicopathological characteristics in osteosarcoma [14], nasopharyngeal carcinoma, NSCLC [15], gastric carcinoma, hepatocellular carcinoma, glioma, cervical cancer, colorectal cancer, and triple-negative breast cancer [16-22]. Therefore, this study hopes to evaluate the potential value of SNHG12 as a prognostic molecular marker through the TCGA database and meta-analysis.

## Methods

### The TCGA database

We analyzed 9497 samples with SNHG12 expression in the TCGA database through the GEPIA tool and plotted the survival curve (http://gepia.cancer-pku.cn/index.html).

### Search and selection online

We searched for potential eligible papers in PubMed, Embase, Cochrane Library, Web of Science, Wanfang, and Wipe up to April 1, 2019. The key words searched are as follows: (“SNHG12” OR “SNHG12 lncRNA, human” OR “ASLNC0408”) AND (“cancer” OR “tumor”). We also searched the references of the original articles and manually consulted the relevant supplementary results. We only examined articles written in English.

### Inclusion and exclusion criteria

This study will strictly abide by the PRISMA statement. First, we excluded duplicated articles. For the remaining articles, we first examined the title and abstract of the study. The exclusion criteria were as follows: (1) not related to the long noncoding RNA SNHG12, (2) only detected SNHG12’s composition and functions, (3) lacked clinical data, and (4) written in another language (not English). By contrast, the acceptance criteria were as follows: (1) the expression level of SNHG12 is described in tumors; (2) research objects must be grouped according to the SNHG12 expression levels; (3) a description of the clinical case characteristics, for example, clinical stage, lymphatic metastasis, distant metastasis (DM), tumor size, and overall survival (OS), was provided; and (4) the study was written in English. Finally, we identified ten papers about SNHG12 by the inclusion and exclusion criteria.

### Data extraction

Three people (WX, JQ, and YQ) independently scanned and extracted all useful data from the 10 papers: (1) paper title, paper authors, year, and area of the patients; (2) tumor type, sample, and size; (3) SNHG12 evaluation method; (4) cut-off values; (5) ORs of SNHG12 for the clinicopathological characteristics, including tumor stage, lymphatic metastasis, grade, depth, tumor size, and gender; and (6) follow-up time. Because the original literature did not provide survival information, according to the method described previously, we used graphs from Engauge Digitizer 4.1 software to estimate the prognosis of the survival curve of each article. If only a Kaplan-Meier curve was used, we extracted survival data from the papers and calculated the HR and 95% CI by previously published techniques [23, 24]. The quality evaluation of the 10 papers was performed based on the REMARK guideline [25]. We took 20 items and each item was rated as 1 point. The score range is 0–20. A score ≥ 80 indicates a high quality paper.

### Statistical analysis

Based on the studies documented in the literature, we collected the 95% confidence intervals and HRs associated with patient prognosis from articles identified based on our criteria. We used the O-E combined variance method to calculate the patient survival outcomes by Review Manager [26]. Then, the statistical heterogeneity of the results was analyzed by using a random effect model (chi square test, *P* < 0.1). Otherwise, we used a fixed-effect model (chi square test, *P* > 0.1) [27]. The results of the meta-analysis are displayed on the forest map. We used the Egger test to further evaluate any expected deviations in the publication. In addition, we defined *P* < 0.05 as significant. We performed all statistical analyses using Review Manager 5.3 and Stata 15.

## Results

**Fig. 1:**
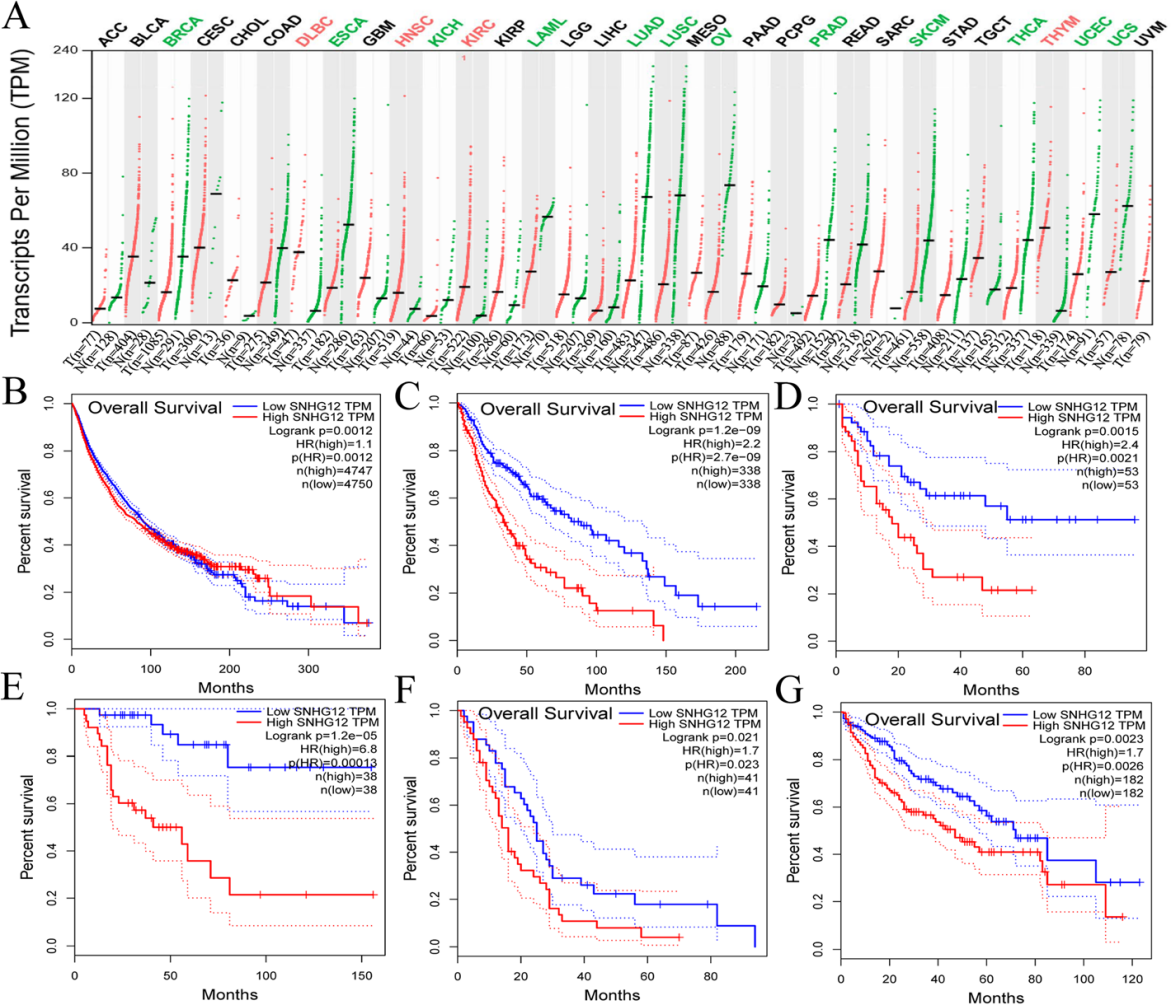
Analysis of clinical samples in the TCGA database. **A** The gene expression profile across all tumor samples and paired normal tissues. **B** Survival curves of all tumors in the TCGA database. **C**–**G** Survival curves of glioma ACC LAML LIHC and MESO

We further validated the expression of SNHG12 and its correlation with prognosis using samples from the TCGA database. In the tumor samples of the TCGA database, the SNHG12 expression level was low in glioma, BRCA, LAML, LUAD, LUSC, and KICH and high in DLBG, HNSC, KIRC, and THYM (Fig. 1A). Survival curves of the tumor samples indicate that SNHG12 is a carcinogenic factor; the higher the expression in the tumor, the worse the prognosis is (Fig. 1B, *P* = 0.0012, HR = 1.1). High expression of SNHG12 in these tumors (glioma, ACC, LAML, LIHC, MESO) was associated with poor prognosis (Fig. 1C–G).

### Analysis of the published studies

#### Characteristics of the included studies

**Fig. 2:**
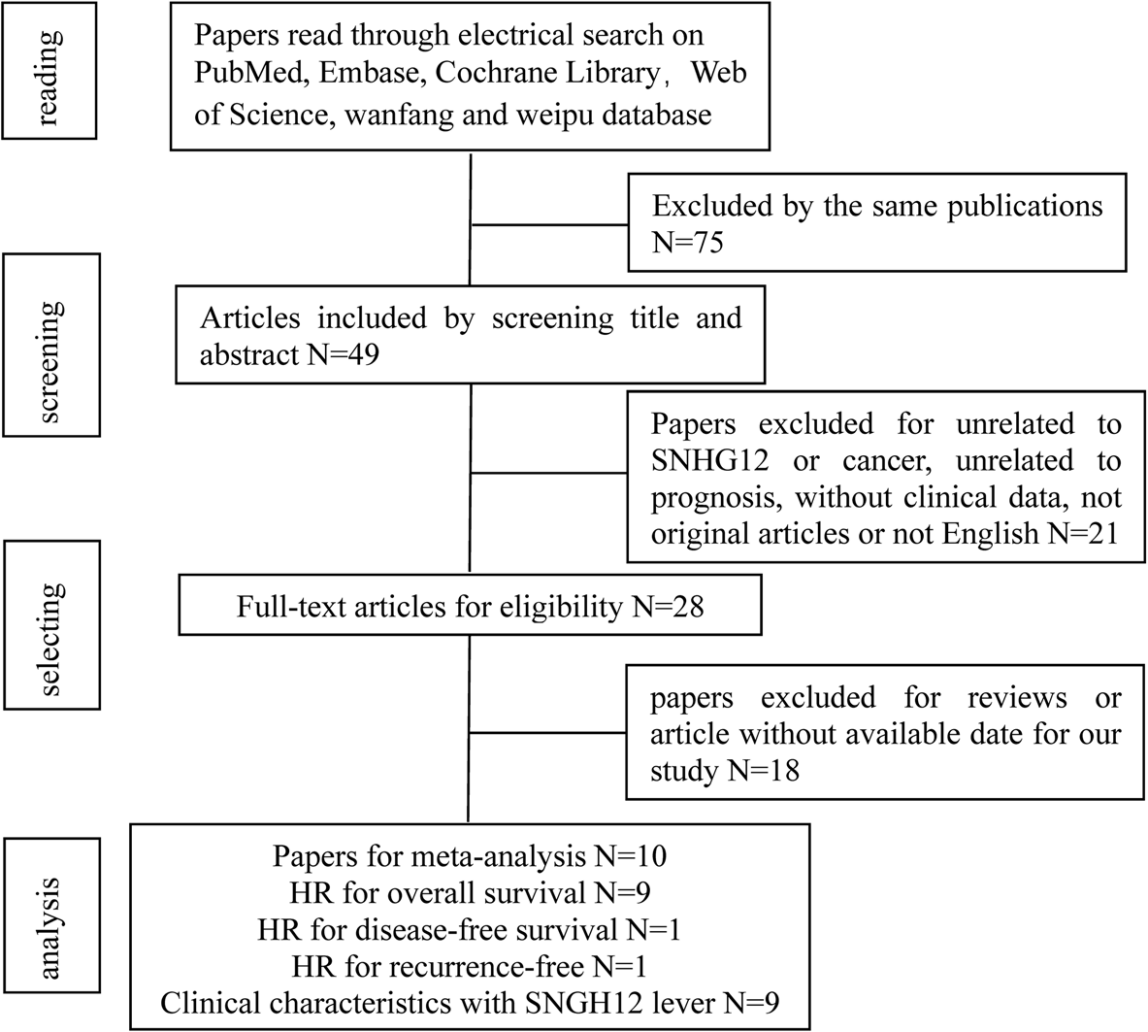
Workflow of searching strategy and study selection in the meta-analysis

In accordance with the previously established standards, we used an electronic browser to select 10 articles by using a combination of free words and subject words from the databases. From 2015 to 2019, there were 679 tissue specimens from 124 records in the 10 articles by reading the full text (Fig. 2). All of the patients in the 10 studies were from Asia and had 9 types of tumors, including nasopharyngeal carcinoma (NPC), colorectal cancer (CRC), cervical cancer (CVC), breast cancer (BC), gastric cancer (GC), hepatocellular cancer (HCC), non-small cell lung carcinoma (NSCLC), osteosarcoma, and glioma (Supplementary Table S1). The main items of the 9 studies are listed in Table 1. To evaluate the relationship between the SNHG12 level and clinicopathological characteristics, we examined 9 valid clinicopathological studies, which included 577 clinical tumor tissues (Supplementary Table S2). According to the REMARK quality evaluation guidelines, we scored the articles in compliance with the evaluation form and divided the articles into grades of 55 to 85% (Supplementary Table S3). We used Engauge Digitizer 4.1 software to extract effect values from the Kaplan-Meier survival curves and calculate the poor HRs and 95% CIs by a previously published method [23].

**Table 1:** The main characteristics of studies with OS included in the prognosis based meta-analysis

Study	Year	Region	Tumor type	Sample size	Specimen	Method	Cut-off value	Follow-up	Outcome	Quality score (%)
Zhou, S [14]	2018	china	osteosarcoma	31	Tissue	qRT-PCR	Mean	60 months	OS	70
Liu, Z [13]	2018	china	NPC	129	Tissue	qRT-PCR	Median	60 months	OS	85
Yang, BF [16]	2018	china	GC	54	Tissue	qRT-PCR	NG	40 months	OS	80
Zhang, HY [17]	2017	china	GC	60	Tissue	qRT-PCR	Median	60 months	OS	75
Lan, T [18]	2017	china	HCC	48	Tissue	qRT-PCR	Median	48 months	OS	80
Lei, W [19]	2018	china	glioma	79	Tissue	qRT-PCR	Median	60 months	OS	85
Wang, JZ [21]	2017	china	CRC	60	Tissue	qRT-PCR	Median	60 months	OS	80
Wang, OC [22]	2017	china	BC	102	Tissue	qRT-PCR	Median	60 months	OS	65
Dong, J [20]	2018	china	CVC	76	Tissue	qRT-PCR	Mean	60 months	OS	80

*NPC* nasopharyngeal carcinoma, *GC* gastric cancer, *HCC* hepatocellular cancer, *CRC* colorectal cancer, *BC* breast cancer, *CVC* cervical cancer, *qRT-PCR* quantitative real-time PCR, *OS* overall survival, *NG* not given

#### Relationship of SNHG12 expression with overall survival in human tumors

**Fig. 3:**
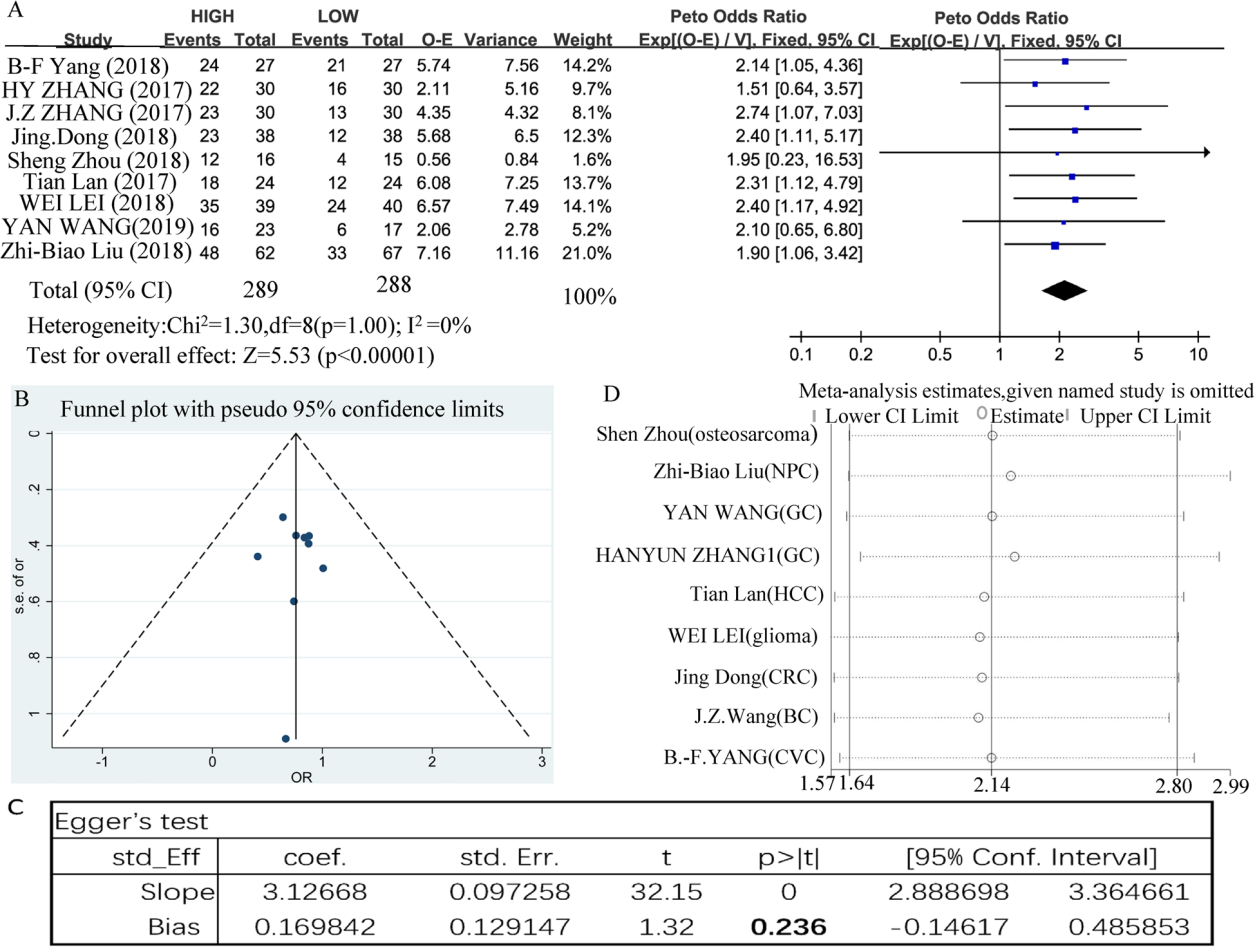
Prognostic value of SNHG12 for OS of cancer patients. **A** Forest plot of HR studies of SNHG12 for OS in a fixed-effect model. Each study is represented by a square and the center of which denotes the HR with a horizontal 95% CI line. The diamond shows the overall OR for combined results. Weights are from a fixed-effect analysis. **B** Funnel plot for potential publication bias in OS analysis. The standard error (SE) of hazard ratio displays a measure of study size on the vertical axis against the hazard ratio on the horizontal axis. **C** Egger’s test for potential publication bias in OS analysis. **D** Sensitivity analysis of the effect of the individual study on the pooled HRs

Nine papers, which included 639 patients, described the relationship between the expression of SNHG12 and OS; the pooled HR was determined to be 2.14 (95% CI 1.63–2.80, *P* < 0.00001) by Review Manager 5.3 software. We recalculated the data in another way through Stata software (HR 2.72, 95% CI 1.95–3.8, *P <* 0.00001). These results indicate that high expression of SNHG12 can significantly reduce the overall survival of patients, suggesting that the prognosis of the patients is poor. The fixed model was adopted because there was no significant heterogeneity (I^2^ = 0, *P* = 1) (Fig. 3**A**). As shown in Table 2, based on tumor type, cut-off values, and sample size, the original articles were grouped differently. According to the analysis of different tumor types, SNHG12 high expression has a significant correlation with poor OS in the patients with digestive system cancer (HR = 2.12, 95% = 1.43–3.15, *P* = 0.0002), nervous system (HR = 2.41, 95% = 1.17–4.97, *P* = 0.02), respiratory system carcinomas (HR = 1.94, 95% = 1.15–3.28, *P* = 0.01), cancers of the reproductive system (HR = 2.40, 95% = 1.12–5.15, *P* = 0.03), and locomotor system (HR = 2.41, 95% = 1.17–4.97, *P* = 0.02). Next, according to the different cut-off scores from the articles, we conducted analysis between different groups, including the median (HR = 2.10, 95% = 1.53–2.89, *P* < 0.00001), and other studies (HR = 2.23, 95% = 1.34–3.71, *P* = 0.002). Then, based on the sample size (≥ 60 or < 60), we divided the studies into two categories. In this analysis, regardless of whether the study had a large sample size (HR = 2.11, 95% = 1.51–2.95, *P* < 0.0001) or a small sample size (HR = 2.19, 95% = 1.39–3.45, *P* = 0.0007), high expression of SNHG12 was an obvious prognostic marker for poor OS. However, we divided the studies into categories based on the sample quality score based on a score of 80. Highly expressed SNHG12 predicts short overall survival for patients from studies with a high quality score (HR = 2.23, 95% = 1.66–3.00, *P* < S0.00001), but the low quality score subgroup did not show significant results due to the combined effects (HR = 1.71, 95% = 0.88–3.32, *P* = 0.11).

**Table 2:** Subgroup and meta-regression analysis of HRs in different cancer type, cut-off, sample size, and quality score subgroup

Subgroup analysis	No. of studies	No. of patients	Pool HR (95% CI)		Weight	Heterogeneity random	
			Fixed	Random		I^**2**^ (%)	***p*** value
Overall survival	9	577	2.17 [1.69, 2.80]	2.17 [1.69, 2.80]	100%	0	0.99
**Cancer type**							
Digestive system	4	222	2.12 [1.43, 3.15]	2.12 [1.43, 3.15]	41.20%	0	0.81
Respiratory system	2	169	1.94 [1.15, 3.28]	1.94 [1.15, 3.28]	23.30%	0	0.88
Reproductive system	1	76	2.40 [1.12, 5.15]	2.40 [1.12, 5.15]	11.00%	-	-
Nervous system	1	79	2.41 [1.17, 4.97]	2.41 [1.17, 4.97]	12.30%	-	-
Locomotor system	1	31	2.41 [1.17, 4.97]	2.41 [1.17, 4.97]	12.30%	-	-
**Cut-off**							
Others	3	161	2.30 [1.51, 3.51]	2.30 [1.51, 3.51]	36.20%	0	0.97
Median	6	416	2.10 [1.53, 2.89]	2.10 [1.53, 2.89]	63.80%	0	0.95
**Quality score**							
Score < 80	3	131	1.71 [0.88, 3.32]	1.71 [0.88, 3.32]	16.50%	0	0.9
Score ≥ 80	6	446	2.23 [1.66, 3.00]	2.23 [1.66, 3.00]	83.50%	0	0.99
**Sample size**							
Size < 60	3	133	2.23 [1.23, 4.03]	2.23 [1.23, 4.03]	20.6%	0	1
Size ≥ 60	6	444	2.12 [1.56, 2.86]	2.12 [1.56, 2.86]	79.4%	0	0.94

The funnel plot and the Egger method were used to test whether the abovementioned combined effects resulted in a publication bias. The meta-results showed that the results are stable without significant asymmetry (Egger’s *P* = 0.236, Fig. 3**B**, **C**). In the same way, we also performed corresponding tests between the different subgroups. The large tumor sample size (Egger’s *P* = 0.326), high quality score (Egger’s *P* = 0.387), and median subgroups (Egger’s *P* = 0.194, Supplementary table 4) were not found to have a publication bias. In addition, by deleting each study and combining the remaining studies in turn, the sensitivity analysis confirmed that the remaining combined HR of the OS was not significantly affected in Fig. 3**D**.

### Association between the SNHG12 level and clinicopathological characteristics

The clinical and pathological characteristics of the included research articles were statistically analyzed, and the results, including the ORs and 95% CIs, are shown in Tables 3, 4, 5, and 6. The summary results show that the expression of SNHG12 is elevated with high stages (OR = 3.94, 95% CI = 2.80–5.53, *P <* 0.00001), lymphatic metastasis (OR = 2.66, 95% CI = 1.65–4.29, *P <* 0.0001), high grade (OR = 2.04, 95% CI = 1.18–3.51, *P* = 0.01), tumor size (OR = 2.79, 95% CI = 1.89–4.14, *P <* 0.00001), and distant metastasis (OR = 2.20, 95% CI = 1.40–3.46, *P* = 0.0006). However, there was no significant difference in patient age (OR = 1.27, 95% CI = 0.93–1.74) or gender (OR = 1.14, 95% CI = 0.78–1.65).

**Table 3:** The subgroup of the relationship and heterogeneity between high SNHG12 expression and tumor stage

Subgroup analysis	No. of studies	No. of patients	Pool OR (95% CI)		Weight	Heterogeneity random	
			Fixed	Random		I^**2**^ (%)	***p*** value
**ORs of tumor stage subgroup**	9	639	3.94 [2.80, 5.53]	3.91 [2.77, 5.51]	100%	0	0.46
**Cancer type**							
Digestive system	4	222	4.54 [2.58, 8.00]	4.54 [2.58, 8.00]	36.60%	0	0.76
Respiratory system	1	129	3.79 [1.75, 8.19]	3.79 [1.75, 8.19]	20.00%	-	-
Reproductive system	2	178	2.24 [1.19, 4.21]	2.24 [1.19, 4.21]	29.70%	0	0.87
Nervous system	1	79	8.79 [3.17, 24.32]	8.79 [3.17, 24.32]	11.40%	-	-
Locomotor system	1	31	10.89 [1.14, 103.98]	10.89 [1.14, 103.98]	2.30%	-	-
**Cut-off**							
Others	3		3.59 [1.83, 7.02]	3.79 [1.58, 9.07]	25.00%	28.5	0.247
Median	6		4.07 [2.74, 6.03]	4.06 [2.73, 6.05]	75.00%	0	0.445
**Sample size**							
Size < 60	4	235	3.76 [2.11, 6.69]	3.69 [2.05, 6.62]	34.70%	0	0.48
Size ≥ 60	5	404	4.04 [2.65, 6.14]	4.07 [2.50, 6.65]	65.30%	23	0.46
**Quality scores**							
Score < 80	4	269	2.70 [1.60, 4.54]	2.62 [1.55, 4.44]	42.70%	0	0.6
Score ≥ 80	5	370	5.25 [3.34, 8.26]	5.25 [3.33, 8.28]	57.30%	0	0.73

**Table 4:** The subgroup of the relationship and heterogeneity between high SNHG12 expression and lymphatic metastasis

Subgroup analysis	No. of studies	No. of patients	Pool HR (95% CI)		Weight	Heterogeneity random	
			Fixed	Random		I^**2**^ (%)	***p*** value
**ORs of lymphatic metastasis subgroup**	5	421	2.60 [1.72, 3.91]	2.66 [1.65, 4.29]	100%	22	0.28
**Cancer type**							
Digestive system	2	114	5.60 [2.44, 12.89]	5.57 [2.41, 12.86]	27.70%	0	0.62
Respiratory system	1	129	1.62 [0.76, 3.45]	1.62 [0.76, 3.45]	27.80%	-	-
Reproductive system	2	178	2.29 [1.23, 4.26]	2.29 [1.23, 4.26]	44.50%	0	0.92
**Cut-off**							
Others	2	130	3.38 [1.60, 7.15]	3.65 [1.16, 11.44]	31.70%	51	0.15
Median	3	291	2.31 [1.41, 3.78]	2.35 [1.35, 4.10]	68.30%	22	0.28
**Sample size**							
Size < 60	2	156	3.29 [1.66, 6.52]	3.65 [1.25, 10.65]	36.30%	50	0.16
Size ≥ 60	3	265	2.27 [1.36, 3.79]	2.31 [1.29, 4.14]	63.70%	19	0.29
**Quality scores**							
Score < 80	3	238	2.73 [1.59, 4.67]	2.73 [1.59, 4.68]	60.50%	0	0.54
Score ≥ 80	2	183	2.42 [1.28, 4.57]	3.09 [0.72, 13.17]	39.50%	73	0.05

**Table 5:** The subgroup of the relationship and heterogeneity between high SNHG12 expression and distant metastasis

Subgroup analysis	No. of studies	No. of patients	Pool HR (95% CI)		Weight	Heterogeneity random	
			Fixed	Random		I^**2**^ (%)	***p*** value
**ORs of distant metastasis subgroup**	6	458	2.20 [1.40, 3.46]	2.23 [1.20, 4.11]	100%	38	0.15
**Cancer type**							
Digestive system	2	120	3.63 [1.68, 7.87]	3.64 [1.57, 8.44]	36.70%	14	0.28
Respiratory system	1	129	1.70 [0.75, 3.85]	1.70 [0.75, 3.85]	25.00%	-	-
Reproductive system	2	178	1.00 [0.39, 2.54]	1.00 [0.39, 2.56]	29.00%	0	0.63
Locomotor system	1	31	8.00 [1.33, 48.18]	8.00 [1.33, 48.18]	9.30%	-	-
**Cut-off**							
Others	2	107	2.43 [0.90, 6.55]	2.82 [0.46, 17.38]	24.50%	64	0.1
Median	4	351	2.14 [1.29, 3.57]	2.14 [1.05, 4.37]	75.50%	43	0.15
**Sample size**							
Size < 60	2	133	1.97 [0.72, 5.33]	2.32 [0.24, 22.63]	23.10%	75	0.04
Size ≥ 60	4	325	2.27 [1.36, 3.77]	2.28 [1.25, 4.15]	76.90%	38	0.15
**Quality scores**							
Score < 80	4	269	2.47 [0.83, 7.30]	2.49 [1.32, 4.69]	48.30%	60	0.06
Score ≥ 80	2	189	1.93 [1.00, 3.70]	1.93 [1.01, 3.70]	51.70%	0	0.61

**Table 6:** The subgroup of the relationship and heterogeneity between high SNHG12 expression and tumor size

Subgroup analysis	No. of studies	No. of patients	Pool OR (95% CI)		Weight	Heterogeneity random	
			Fixed	Random		I^**2**^ (%)	***p*** value
**ORs of tumor size subgroup**	8	510	2.79 [1.89, 4.14]	3.29 [1.66, 6.51]	100%	60	0.01
**Cancer type**							
Digestive system	4	222	5.43 [3.02, 9.76]	5.43 [3.01, 9.79]	52.70%	0	0.8
Reproductive system	2	178	0.91 [0.40, 2.09]	1.01 [0.30, 3.43]	21.90%	26	0.24
Locomotor system	1	31	8.80 [1.69, 45.76]	8.80 [1.69, 45.76]	9.60%	-	-
Nervous system	1	79	1.58 [0.65, 3.84]	1.58 [0.65, 3.84]	15.90%	-	-
**Cut-off**							
Others	3	161	1.96 [1.05, 3.66]	2.82 [0.58, 13.63]	37.90%	79	0.008
Median	5	349	3.53 [2.12, 5.88]	3.73 [1.92, 7.24]	62.10%	33	0.2
**Sample size**							
Size < 60	4	235	4.89 [2.36, 10.14]	4.92 [2.37, 10.19]	41.30%	0	0.87
Size ≥ 60	4	275	2.18 [1.36, 3.50]	2.52 [0.86, 7.42]	58.70%	78	0.003
**Quality scores**							
Score < 80	4	269	2.13 [1.18, 3.82]	2.83 [0.79, 10.10]	45.40%	71	0.02
Score ≥ 80	4	241	3.51 [2.06, 5.99]	3.83 [1.75, 8.41]	54.60%	49	0.12

Tumor size (I^2^ = 60%, *P* = 0.01) showed obvious statistical heterogeneity in 8 studies (Table 6). The existence of heterogeneity in each subgroup was shown in the random effects model. In the tumor size heterogeneity analysis, we discovered notable heterogeneity in the low quality score subgroup (I^2^ = 71, *P* = 0.02), the large sample size subgroup (I^2^ = 78%, *P* = 0.003), and other the cut-off subgroups (I^2^ = 79, *P* = 0.008). According to the three subgroups, we found that the Jing Dong study (HR = 0.72, 95% CI = 0.29–1.81) was in the above three subgroups and may be the main source of heterogeneity. No proof of statistical heterogeneity was found in lymphatic metastasis subgroup, the tumor stage subgroup and distant metastasis subgroup (I^2^ = 22%, *P* = 0.28; I^2^ = 0%, *P* = 0.46; and I^2^ = 38%, *P* = 0.15). However, Zhi-Biao Liu (OR = 1.62, 95% = 0.76–3.45) showed no clinical significance or obvious heterogeneity by stratification analysis of the lymphatic quality subgroup. Further investigation of heterogeneity in distant metastasis revealed obvious heterogeneity in the low quality score (HR = 2.47, 95% CI = 0.83–7.30, *P* > 0.01, I^2^ = 60%, *P* = 0.06) subgroups and tumor size (OR = 2.32, 95% CI = 0.24–22.63, *P* > 0.01, I^2^ = 75%, *P* = 0.04).

We are concerned about publication bias in the statistical analysis of clinicopathological characteristics. We performed the Egger linear regression test. The results showed that there were no published biases in lymph node metastasis, the tumor stage, metastasis and size subgroups (Egger’s *P* = 0.238, Egger’s *P* = 0.192, Egger’s *P* = 0.149, and Egger’s *P* = 0.097) (Supplementary Table 5). Because of deleting each study and the remaining combined HR of the OS not significantly affected, sensitivity analysis of different subgroups showed stable results in Supplementary Figure 1.

## Discussion

According to the GBD study, about 20% men and 16.7% women will get tumor, and 12.5% men and 9% women will die because of tumor in the worldwide. Compared with that in other regions of the world, Asian countries have the highest cancer mortality rate due to the limitations of existing medical conditions and technical levels [1]. Although, cancer treatment has made significant progress, the long-term overall survival and high quality of life are still major challenges in medicine. Therefore, new molecular biomarkers are urgently needed in clinical practice to promote extensive clinical screening, improve the prognosis of tumor patients, and provide potential therapeutic targets [28–30].

Sequencing technology has helped reveal genomic information and has shown that more than 90% of noncoding RNAs are considered “transcriptional noise” and are nonessential. Further in-depth studies have shown that these findings are no longer valid for various processes. Many recent studies have confirmed that lncRNAs regulate the transcription and translation of genes involved in tumor cells through a variety of pathways, ultimately affecting tumor biological behavior. Due to the heterogeneity of tumors and the complexity of the tumor microenvironment, these molecules can play a carcinogenic or tumor suppressive role in the same tumor. There is evidence that lncRNAs can act as molecular scaffolds, sponges, or coactivators in tumor scaffolds by interacting with DNA, RNA, or proteins [5, 31, 32]. At present, increasing evidence has shown that the abnormally high expression of SNHG12 plays a carcinogenic role in various malignant tumors [13–22, 12, 33, 11, 10].

Further research has shown that overexpression of SNHG12 is associated with rapid cancer proliferation, strong invasion and migration, and high rates of metastasis, recurrence, and chemical resistance [31, 32, 34] (Table 7). Moreover, in lung cancer, this molecule can inhibit metastasis and epithelial-mesenchymal transition when inhibited [15]. Furthermore, increased SNHG12 not only regulates malignant behavior by activating oncogenes (AMOT, HuR slug/ZEB2) [15, 35], signaling pathways (MAPK/Slug, wnt/β-catenin, and Notch signaling pathway) [13, 14, 10, 34, 36], and the microRNA-gene axis (miR-125b/STAT3 and miR-101-3p/FOXP1) [33, 31, 37] but also enhances chemoresistance of tumor cells [31, 32]. SNHG12 is negative regulation miR-195-5p, miR-320, miR-129, miR-193a-3p, microRNA-199a/b-5p, and miR-424-5 [14, 17, 18, 20, 12]. Yin et al., Zhao et al., and Long et al. found that SNHG12 regulates brain microvascular endothelial cell death, the inflammatory response, and angiogenesis during and after ischemic stroke through target genes and signaling pathways (Sirt1, AMPK signaling pathway, and miR-150/VEGF pathway) [38–40]. In addition, the expression level of SNHG12 in gastric cancer, osteoma, colon cancer, hepatocellular carcinoma, and various other tumors is related to the clinical features and prognosis of cancer patients. Clinicians should fully consider the situation of patients in the early stage of treatment to provide an appropriate treatment plan and reasonable results for the patients and the patient’s family members, which will minimize the expectations between doctors and patients.

**Table 7:** The relationship between microRNA and SNHG12 in the occurrence and development of cancer

microRNAS	Cancer type	Interaction with SNHG12	Correlation with SNHG12	Potential target genes	Function	References
miRNA-129	Ovarian cancer	Direct binging	Negative	SOX4	Cell proliferation and migration	[12]
microRNA-195	Prostate cancer	Direct binging	Negative	Wnt/β-catenin	Cell proliferation	[36]
miR-218	NSCLC	Direct binging	Negative	MMP9 E-cadherin vimentin caspase3 caspase9 slug ZEB2	Cell proliferation, invasion, and migration	[15]
miR-125b	Cervical cancer	Direct binging	Negative	stat3	Cell proliferation migration and invasion	[37]
miR-101-3p	Glioma	Direct binging	Negative	FOXP1	Proliferation AND apoptosis	[33]
microRNA-199a/b-5p	Gastric carcinoma	Direct binging	Negative	-	Cell proliferation	[16]
MiR-424-5p	Cervical cancer	Direct binging	Negative	-	Cell growth and invasion	[20]
miR-195	Glioma	Direct binging	Negative	SOX5 TDP43	Cell proliferation, migration, and invasion	[11]
miR-195-5p	Osteosarcoma	Direct binging	Negative	Notch2 CCND1 CDK6 CDK4 NICD HES1	Cell proliferation, invasion, and migration	[14]
miR-320	Gastric cancer	Direct binging	Negative	AKT ERK CRKL	Cell proliferation and invasion	[17]
miR-199a/b-5p	Hepatocellular carcinoma	Direct binging	Negative	Erk1 MLK3 IκB NF-κB	Cell proliferation invasion and migration	[18]
miR-320a	Osteosarcoma	Direct binging	Negative	MCL1	Doxorubicin resistance	[31]
miR-181a	NSCLC	Direct binging	Negative	MAPK Slug	Multidrug resistance	[34]
miR-199a	Ischemic stroke	Direct binging	Negative	MCP1 IL-6 VEGFA and FGFb	Death, the inflammatory response, and promote angiogenesis	[38]
miR-150	Oschemic stroke	Direct binging	Negative	VEGF	Angiogenesis	[40]
miR-199a	Cerebral ischemia	Direct binging	Negative	AMPK SIRT1	Attenuate cerebral ischemia injury	[39]

Here, we wanted to observe whether the role of SNHG12 in tumors is the same, that is, to suppress or promote cancer. First, we found that SNHG12 can play a role in promoting tumors in different tumors via the GEPIA. Second, the meta-analysis showed that high SNHG12 expression may be considered a negative factor for prognosis of patients with various tumors (*P* < 0.00001). Furthermore, the aggregated data showed that SNHG12 high expression was associated with tumor stage (*P* < 0.00001), poor cancer outcome (*P* = 0.01), positive lymph node metastases (*P* < 0.00001), distant metastases (*P* = 0.0006), and tumor size (*P* < 0.00001). However, whether using the TCGA database or meta-analyses, there are limitations that cannot be ignored. First, most of the sample sources in the TCGA database and research articles are geographically limited. There are many regions in the world, the geographical environment and customs are different, and the diversity of the samples cannot be ignored. Second, the lack of statistical analysis of the raw data in some articles resulted in heterogeneity in the HR and OR in the later statistical analyses. Third, because different articles have different classifications and qualitative characteristics of the original data, different standards are an important reason for the heterogeneity of the results. At last, in these studies, Liu et al. used univariate and multivariate methods to analyze the survival data of the NPC patients; others only used the univariate method to assess the survival data and did not discuss the relationship between multiple factors.

## Conclusions

The current analysis shows that, whether in the TCGA database or in published articles, overexpression of SNHG12 is closely related to the poor prognosis of tumor patients. SNHG12 high expression is related to the OS, tumor stage, lymph node metastasis, tumor size, distant metastasis, and high tumor grade, especially in the Chinese population. Therefore, SNHG12 can be used as an effective biomarker to predict the prognosis and tumor progression of cancer patients. However, larger and well-designed studies are needed to confirm the results of this analysis.

## Supplementary Information

**Additional file 1 **: **Supplementary Table S1**. The cancer type of the 10 study papers.

**Additional file 2 **: **Supplementary Table S2**. The clinicopathological characteristics of 9 study in this paper.

**Additional file 3 **: **Supplementary Table S3**. The value of quality evaluation estimated on the REMARK guideline.

**Additional file 4 **: **Supplementary Table S4**. The Egger’s test of different subgroup.

**Additional file 5 **: **Supplementary Table S5**. The Egger’s test of different subgroup.

**Additional file 6 **: **Supplementary Figure 1**. Sensitivity analysis of the effect of the individual subgroup on the pooled ORs.

## Data Availability

All data generated or analyzed during this study are included in this published article.

## Abbreviations

GBD: Global burden of disease
lncRNA: Long noncoding RNA
SNHG12: Small nucleolar RNA host gene 12
OS: Overall survival
DM: Distant metastasis
HR: Hazard ratio
OR: Odds ratios
CI: Confidence interval
miRNAs: MicroRNAs
BRCA: Breast invasive carcinoma
HNSC: Head and Neck squamous cell carcinoma
LAML: Acute Myeloid Leukemia
LUSC: Lung squamous cell carcinoma
LIHC: Liver hepatocellular carcinoma
LUAD: Lung adenocarcinoma
MESO: Mesothelioma
NSCLC: Non-small cell lung carcinoma
GC: Gastric cancer
CRC: Colorectal cancer
KIRC: Kidney renal clear cell carcinoma
HCC: Hepatocellular cancer
BC: Breast cancer
CVC: Cervical cancer
NPC: Nasopharyngeal carcinoma
REMARK: Reporting recommendations for tumor marker prognostic studies

## References

[1] Bray F, Ferlay J, Soerjomataram I, Siegel RL, Torre LA, Jemal A (2018). Global cancer statistics 2018: GLOBOCAN estimates of incidence and mortality worldwide for 36 cancers in 185 countries. CA Cancer J Clin.

[2] Hanahan D, Weinberg RA (2011). Hallmarks of cancer: the next generation. Cell.

[3] Ponting CP, Oliver PL, Reik W (2009). Evolution and functions of long noncoding RNAs. Cell.

[4] Gutschner T, Diederichs S (2012). The hallmarks of cancer: a long non-coding RNA point of view. RNA Biol.

[5] He RZ, Luo DX, Mo YY (2019). Emerging roles of lncRNAs in the post-transcriptional regulation in cancer. Genes Dis.

[6] Huang Y, Liu N, Wang JP, Wang YQ, Yu XL, Wang ZB, Cheng XC, Zou Q (2012). Regulatory long non-coding RNA and its functions. J Physiol Biochem.

[7] Nagano T, Fraser P (2011). No-nonsense functions for long noncoding RNAs. Cell.

[8] Zhang Y, Zhang L, Wang Y, Ding H, Xue S, Qi H, Li P (2019). MicroRNAs or long noncoding RNAs in diagnosis and prognosis of coronary artery disease. Aging Dis.

[9] Zhai W, Li X, Wu S, Zhang Y, Pang H, Chen W (2015). Microarray expression profile of lncRNAs and the upregulated ASLNC04080 lncRNA in human endometrial carcinoma. Int J Oncol.

[10] Ding S, Qu W, Jiao Y, Zhang J, Zhang C, Dang S (2018). LncRNA SNHG12 promotes the proliferation and metastasis of papillary thyroid carcinoma cells through regulating wnt/beta-catenin signaling pathway. Cancer Biomark.

[11] Liu X, Zheng J, Xue Y, Qu C, Chen J, Wang Z, Li Z, Zhang L, Liu Y (2018). Inhibition of TDP43-mediated SNHG12-miR-195-SOX5 feedback loop impeded malignant biological behaviors of glioma cells. Mol Ther Nucleic Acids.

[12] Sun D, Fan XH (2019). LncRNA SNHG12 accelerates the progression of ovarian cancer via absorbing miRNA-129 to upregulate SOX4. Eur Rev Med Pharmacol Sci.

[13] Liu ZB, Tang C, Jin X, Liu SH, Pi W (2018). Increased expression of lncRNA SNHG12 predicts a poor prognosis of nasopharyngeal carcinoma and regulates cell proliferation and metastasis by modulating Notch signal pathway. Cancer Biomark.

[14] Zhou S, Yu L, Xiong M, Dai G (2018). LncRNA SNHG12 promotes tumorigenesis and metastasis in osteosarcoma by upregulating Notch2 by sponging miR-195-5p. Biochem Biophys Res Commun.

[15] Wang YAN, Liang S, Yu Y, Shi Y, Zheng H (2019). Knockdown of SNHG12 suppresses tumor metastasis and epithelial-mesenchymal transition via the slug/ZEB2 signaling pathway by targeting miR-218 in NSCLC. Oncol Lett.

[16] Yang BF, Cai W, Chen B. LncRNA SNHG12 regulated the proliferation of gastric carcinoma cell BGC-823 by targeting microRNA-199a/b-5p. Eur Rev Med Pharmacol Sci. 2018;22(5):1297–306. 10.26355/eurrev_201803_14471. PMID: 2955487. 10.26355/eurrev_201803_14471 29565487

[17] Zhang H, Lu W (2018). LncRNA SNHG12 regulates gastric cancer progression by acting as a molecular sponge of miR-320. Mol Med Rep.

[18] Lan T, Ma W, Hong Z, Wu L, Chen X, Yuan Y. Long non-coding RNA small nucleolar RNA host gene 12 (SNHG12) promotes tumorigenesis and metastasis by targeting miR-199a/b-5p in hepatocellular carcinoma. J Exp Clin Cancer Res. 2017;36(1):11. Published 2017 Jan 10. 10.1186/s13046-016-0486-9. 10.1186/s13046-016-0486-9 PMC5223416 28073380

[19] Lei W, Wang ZL, Feng HJ, Lin XD, Li CZ, Fan D (2018). Long non-coding RNA SNHG12 promotes the proliferation and migration of glioma cells by binding to HuR. Int J Oncol.

[20] Dong J, Wang Q, Li L, Zhang XJ (2018). Upregulation of long non-coding RNA small nucleolar RNA host gene 12 contributes to cell growth and invasion in cervical cancer by acting as a sponge for MiR-424-5p. Cell Physiol Biochem.

[21] Wang JZ, Xu CL, Wu H, Shen SJ. LncRNA SNHG12 promotes cell growth and inhibits cell apoptosis in colorectal cancer cells. Braz J Med Biol Res. 2017;50(3):e6079. 10.1590/1414-431X20176079. PMID: 28225893; PMCID: PMC5333723. 10.1590/1414-431X20176079 PMC5333723 28225893

[22] Wang O (2017). C-MYC-induced upregulation of lncRNA SNHG12 regulates cell proliferation, apoptosis and migration in triple-negative breast cancer.

[23] Tierney JF, Stewart LA, Ghersi D, Burdett S, Sydes MR (2007). Practical methods for incorporating summary time-to-event data into meta-analysis. Trials.

[24] Parmar MK, Torri V, Stewart L. Extracting summary statistics to perform meta-analyses of the published literature for survival endpoints. Stat Med. 1998;17(24):2815–34. 10.1002/(SICI)1097-0258(19981230)17:24<2815::AID-SIM110>3.0.CO;2-8. 10.1002/(sici)1097-0258(19981230)17:24<2815::aid-sim110>3.0.co;2-8 9921604

[25] Altman DG, McShane LM, Sauerbrei W, Taube SE (2012). Reporting Recommendations for Tumor Marker Prognostic Studies (REMARK): explanation and elaboration. PLoS Med.

[26] Teng Li, Yi Xing, Shu-cheng Liu, Xiao-min Han, Wen-cheng Li, Min Chen. Long-term versus short-term introvesical chemotherapy in patients with non-muscle-invasive bladder cancer: A systematic review and meta-analysis of the published results of randomized clinical trials. J Huazhong Univ Sci Technolog Med Sci. 2014;34(5):706–15. 10.1007/s11596-014-1340-y 25318881

[27] DerSimonian R, Laird N. Meta-analysis in clinical trials. Control Clin Trials. 1986;7(3):177–88. 10.1016/0197-2456(86)90046-2 3802833

[28] Burke HB (2016). Predicting clinical outcomes using molecular biomarkers. Biomark Cancer.

[29] Kunej T, Obsteter J, Pogacar Z, Horvat S, Calin GA (2014). The decalog of long non-coding RNA involvement in cancer diagnosis and monitoring. Crit Rev Clin Lab Sci.

[30] Sarfi M, Abbastabar M, Khalili E (2019). Long noncoding RNAs biomarker-based cancer assessment. J Cell Physiol.

[31] Zhou B, Li L, Li Y, Sun H, Zeng C (2018). Long noncoding RNA SNHG12 mediates doxorubicin resistance of osteosarcoma via miR-320a/MCL1 axis. Biomed Pharmacother.

[32] Zhu L, Zhang X, Fu X, Li Z, Sun Z, Wu J, Wang X, Wang F, Li X, Niu S (2019). c-Myc mediated upregulation of long noncoding RNA SNHG12 regulates proliferation and drug sensitivity in natural killer/T-cell lymphoma.

[33] Sun Y, Liu J, Chu L, Yang W, Liu H, Li C, Yang J (2018). Long noncoding RNA SNHG12 facilitates the tumorigenesis of glioma through miR-101-3p/FOXP1 axis. Gene.

[34] Wang P, Chen D, Ma H, Li Y (2017). LncRNA SNHG12 contributes to multidrug resistance through activating the MAPK/Slug pathway by sponging miR-181a in non-small cell lung cancer. Oncotarget.

[35] Ruan W, Wang P, Feng S, Xue Y, Li Y (2016). Long non-coding RNA small nucleolar RNA host gene 12 (SNHG12) promotes cell proliferation and migration by upregulating angiomotin gene expression in human osteosarcoma cells. Tumour Biol.

[36] Song J, Wu X, Ma R, Miao L, Xiong L, Zhao W. Long noncoding RNA SNHG12 promotes cell proliferation and activates Wnt/β-catenin signaling in prostate cancer through sponging microRNA-195. J Cell Biochem. 2019;120(8):13066–75. 10.1002/jcb.28578. Epub 2019 Apr 4. PMID: 30945357. 10.1002/jcb.28578 30945357

[37] Jin XJ, Chen XJ, Zhang ZF, Hu WS, Ou RY, Li S, Xue JS, Chen LL, Hu Y, Zhu H. Long noncoding RNA SNHG12 promotes the progression of cervical cancer via modulating miR-125b/STAT3 axis. J Cell Physiol. 2019;234(5):6624–32. 10.1002/jcp.27403. Epub 2018 Sep 24. PMID: 30246459. 10.1002/jcp.27403 30246459

[38] Yin WL, Yin WG, Huang BS, Wu LX (2019). LncRNA SNHG12 inhibits miR-199a to upregulate SIRT1 to attenuate cerebral ischemia/reperfusion injury through activating AMPK signaling pathway. Neurosci Lett.

[39] Long FQ, Su QJ, Zhou JX, Wang DS, Li PX, Zeng CS, Cai Y (2018). LncRNA SNHG12 ameliorates brain microvascular endothelial cell injury by targeting miR-199a. Neural Regen Res.

[40] Zhao M, Wang J, Xi X, Tan N, Zhang L (2018). SNHG12 promotes angiogenesis following ischemic stroke via regulating miR-150/VEGF pathway. Neuroscience.

