# Supplementary material for: Long noncoding RNA SNHG12 is a potential diagnostic and prognostic biomarker in various tumors
Source: Chin Neurosurg J. 2021 Aug 9;7:37. doi: 10.1186/s41016-021-00250-4 (PMC8351140; doi:10.1186/s41016-021-00250-4)

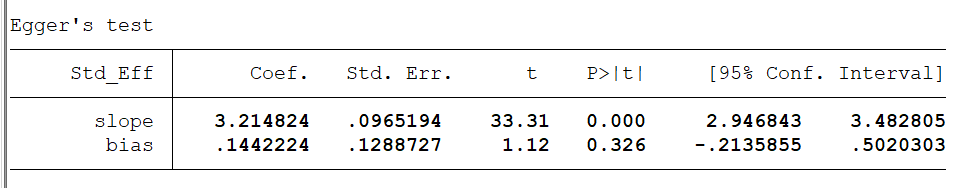
**Supplementary Table S4: the Egger’s test of different subgroup**

**1.Sample size ≥60**

**2.Quality scores ≥80**

**3.median group**


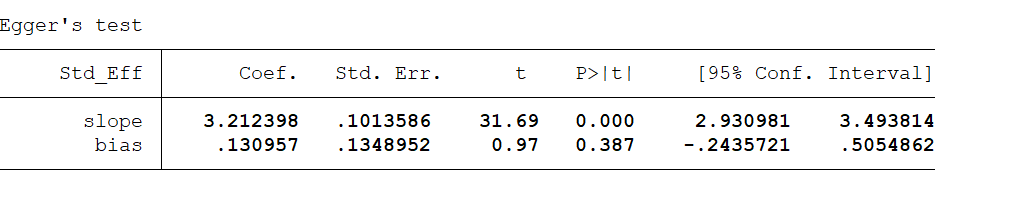


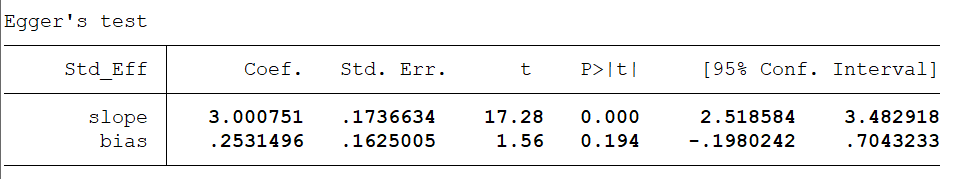

Supplement: Supplementary file 4 — Additional file 4 : Supplementary Table S4. The Egger’s test of different subgroup. [file 41016_2021_250_MOESM4_ESM.docx]
