# Supplementary material for: Long noncoding RNA SNHG12 is a potential diagnostic and prognostic biomarker in various tumors
Source: Chin Neurosurg J. 2021 Aug 9;7:37. doi: 10.1186/s41016-021-00250-4 (PMC8351140; doi:10.1186/s41016-021-00250-4)
Supplement: Supplementary file 5 — Additional file 5 : Supplementary Table S5. The Egger’s test of different subgroup. [file 41016_2021_250_MOESM5_ESM.docx]

**Supplementary Table S5: the Egger’s test of different subgroup**

**1. tumor stage subgroup**


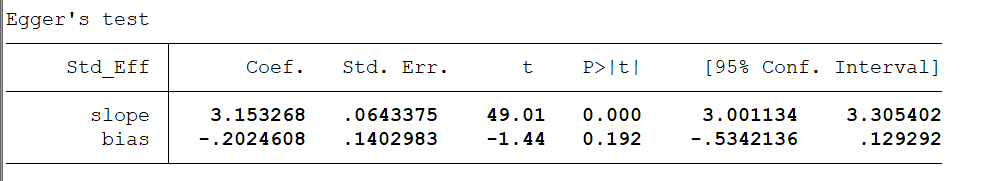


**2. distant metastasis subgroup**


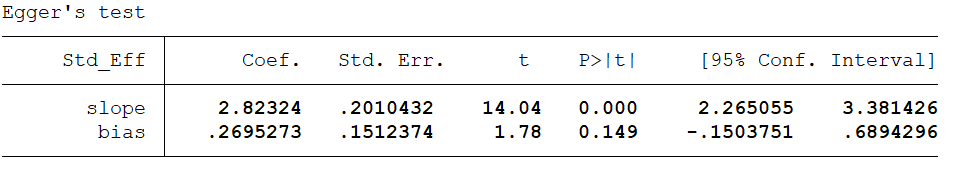


**3. Lymphatic metastasis subgroup**


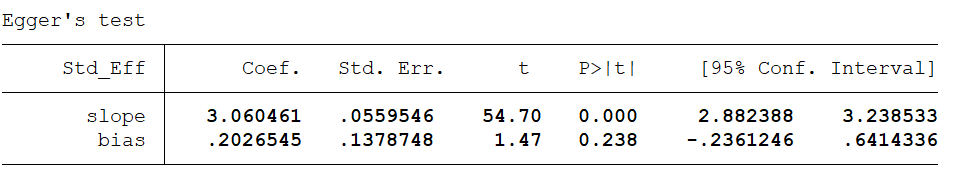


**4.** **tumor size subgroup**
