# Supplementary material for: Long noncoding RNA SNHG12 is a potential diagnostic and prognostic biomarker in various tumors
Source: Chin Neurosurg J. 2021 Aug 9;7:37. doi: 10.1186/s41016-021-00250-4 (PMC8351140; doi:10.1186/s41016-021-00250-4)
Supplement: Supplementary file 6 — Additional file 6 : Supplementary Figure 1. Sensitivity analysis of the effect of the individual subgroup on the pooled ORs. [file 41016_2021_250_MOESM6_ESM.docx]

**Supplementary Figure1：Sensitivity analysis of the effect of the individual subgroup on the pooled ORs.**


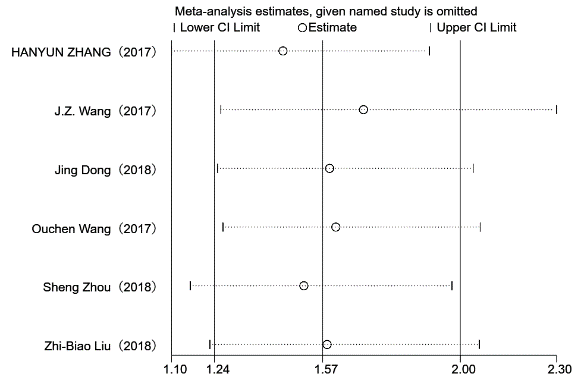

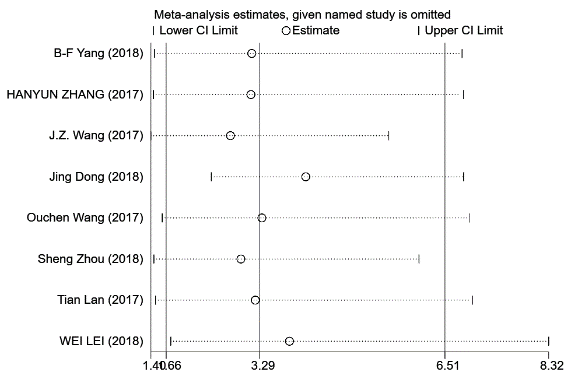
**A B**


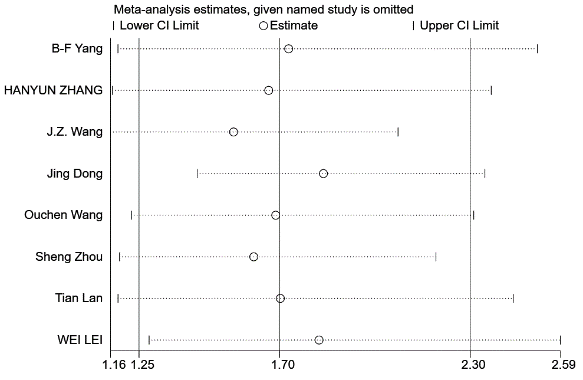

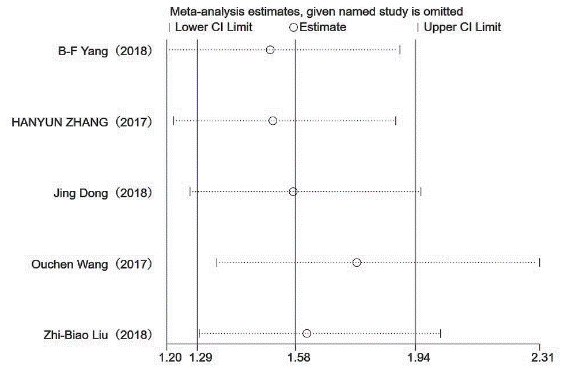


**C D**

**A：Sensitivity analysis of tumor stage subgroup. B：Sensitivity analysis of distant metastasis subgroup.**

**C：Sensitivity analysis of Lymphatic metastasis subgroup. D：Sensitivity analysis of tumor size subgroup.**
